# End-of-Life Care Decision-Making in Stroke

**DOI:** 10.3389/fneur.2021.702833

**Published:** 2021-09-28

**Authors:** Lucy Gao, Charlie W. Zhao, David Y. Hwang

**Affiliations:** ^1^Yale School of Medicine, New Haven, CT, United States; ^2^Division of Neurocritical Care and Emergency Neurology, Yale School of Medicine, New Haven, CT, United States

**Keywords:** stroke, end-of-life, palliative care, goals-of-care, advance care planning, surrogate decision-maker, shared decision-making

## Abstract

Stroke is one of the leading causes of death and long-term disability in the United States. Though advances in interventions have improved patient survival after stroke, prognostication of long-term functional outcomes remains challenging, thereby complicating discussions of treatment goals. Stroke patients who require intensive care unit care often do not have the capacity themselves to participate in decision making processes, a fact that further complicates potential end-of-life care discussions after the immediate post-stroke period. Establishing clear, consistent communication with surrogates through shared decision-making represents best practice, as these surrogates face decisions regarding artificial nutrition, tracheostomy, code status changes, and withdrawal or withholding of life-sustaining therapies. Throughout decision-making, clinicians must be aware of a myriad of factors affecting both provider recommendations and surrogate concerns, such as cognitive biases. While decision aids have the potential to better frame these conversations within intensive care units, aids specific to goals-of-care decisions for stroke patients are currently lacking. This mini review highlights the difficulties in decision-making for critically ill ischemic stroke and intracerebral hemorrhage patients, beginning with limitations in current validated clinical scales and clinician subjectivity in prognostication. We outline processes for identifying patient preferences when possible and make recommendations for collaborating closely with surrogate decision-makers on end-of-life care decisions.

## Introduction: Epidemiology of Life-Sustaining Therapy for Severe Stroke Patients

Stroke is a leading cause of death and long-term disability in the United States (US) (1, 2). The term “stroke” for this review focuses on two subtypes: acute ischemic stroke (AIS) and intracerebral hemorrhage (ICH). Clinicians are often confronted with issues related to end-of-life (EOL) care for stroke patients, such as code status, dysphagia care, and airway management (3). In order to tailor these decisions to patients' wishes, goals-of-care (GOC) discussions regarding acceptable quality of life (QoL) that require collaboration with surrogate decision-makers of incapacitated patients are needed.

Code status changes are among the earliest decisions that may occur during hospitalization for severe stroke. In practice, do-not-resuscitate (DNR) orders are often placed as early as within 24 hours of emergency department admission for both ICH (4) and AIS (5) patients. Approximately 13–26% of stroke patients receive DNR orders within 24 hours of admission (4, 5), with higher proportions of DNR status among those who later die of stroke (6, 7). There is concern that the act of making a patient DNR by itself affects clinicians' impressions of prognosis and independently increases the likelihood of mortality in AIS (5) and ICH (8, 9). This possible “self-fulfilling prophecy” is a well-established concern in stroke care (10).

In the days to weeks after admission, issues of nutrition and airway management often come to the forefront of decision-making. Percutaneous endoscopic gastrostomy (PEG) placement is currently performed throughout the US in 8.8% of patients with AIS (11) and 10.4% for ICH (12), with variation amongst institutions (11, 12). Over half of PEG placements for AIS occur in the first week of admission (13). For stroke patients who have difficulty maintaining an open airway or who require prolonged mechanical ventilation, tracheostomy in the US is commonly performed 6–14 days after stroke onset (14, 15), with increasing numbers over the past two decades (14). Rates of life-sustaining interventions are higher in minority patients than white patients (16), including PEG (17, 18) and tracheostomy (18).

In conjunction with these decisions, surrogates and clinical teams often decide to forgo life-sustaining measures and instead pursue comfort measures only (CMO). Withdrawal of life-sustaining therapy (WLST) is more common in neuro-intensive care units (Neuro-ICUs) than medical intensive care units (MICUs) (19), with up to 26% of all ICH patients in one single-center series undergoing WLST (20). Almost half of all stroke deaths occur inpatient (21), and hospitalized stroke patients have extensive palliative care needs (22, 23) that may not always be met. In one single-center US study from 2009-2015, about 4% of AIS patients were discharged to hospice (22).

In this brief review, we discuss the issues that arise when making EOL care decisions regarding stroke patients. We discuss prognostication tools, their limitations, methods to determine an incapacitated patient's wishes including advance care planning documentation (ACP) and best practices for shared decision-making with surrogates.

## Prognostication: Limitations of Clinical Scales

One factor in EOL decision-making involves prognostication of long-term outcome or natural disease history. Multiple clinical scales have been developed to predict mortality and functional outcome after stroke (24–26), several of which have been externally validated (Table 1).

**Table 1:** Selected clinical scales developed for acute ischemic stroke and intracerebral hemorrhage.

**Scale**	**Original study**	**Predictors**	**Outcome variables**
**Acute Ischemic Stroke**			
THRIVE	Flint et al., 2010 (27)	NIHSS score, age, presence of hypertension, diabetes, atrial fibrillation	Mortality and mRS 90 days after stroke with endovascular treatment
iScore	Saposnik et al., 2011 (28)	Age, sex, stroke severity, stroke subtype, comorbid conditions, preadmission level of function, glucose on admission	Death at 30 days or mRS = 3–5 at discharge/Death at 30 days or institutionalization at discharge
DRAGON	Strbian et al., 2012 (29)	Early infarct signs on admission CT, pre-stroke mRS, age, baseline glucose, onset to treatment time, baseline NIHSS	mRS 3 months after stroke treated with IV tPA
SOAR	Myint et al., 2014 (30)	Age, gender, ischemic vs hemorrhagic stroke, vascular territory, pre-stroke mRS	Inpatient and 7-day mortality
**Intracerebral hemorrhage**			
ICH score	Hemphill et al., 2001 (31)	GCS score, age, infratentorial origin, ICH volume, IVH	30-day mortality
Modified ICH score	Cheung and Zou, 2003 (32)	NIHSS, age, infratentorial origin, ICH volume, IVH	30-day mortality or mRS score ≤ 2
New ICH score	Cheung and Zou, 2003 (32)	NIHSS, temperature, pulse pressure, IVH, subarachnoid extension	30-day mortality or mRS score ≤ 2
ICH-GS	Ruiz-Sandoval et al., 2007 (33)	GCS score, age, ICH location, ICH volume, IVH	30-day mortality and GOS ≥ 4
FUNC	Rost et al., 2008 (34)	Age, GCS score, ICH location, ICH volume, pre-ICH cognitive impairment	90-day GOS ≥ 4
max-ICH	Sembill et al., 2017 (35)	ICH volume, age, NIHSS, IVH, oral anticoagulation	12-month mortality and mRS score in maximally treated patients

*GCS, Glasgow Coma Scale; GOS, Glasgow Outcome Score; ICH, intracerebral hemorrhage; IVH, intraventricular hemorrhage; mRS, modified Rankin scale; NIHSS, National Institute of Health Stroke Scale; tPA, tissue plasminogen activator*.

Common predictor variables in AIS scales include age, stroke severity, pre-stroke functional status, comorbidities, and stroke subtype (24), with some scales utilizing imaging characteristics (36).

For ICH, many prognostication scales are based on variations of the “original” ICH score (33, 37–40), which was initially published with 30-day mortality data utilizing age, Glasgow Coma Scale at admission, ICH location, ICH volume, and presence of intraventricular hemorrhage (31).

Some published data suggest that scales largely outperform the “subjective” opinion of clinicians at predicting mortality and functional disability (41–43). However, these studies generally involved asking clinicians to prognosticate expected outcomes from hypothetical patient vignettes, which simplify and distill information that would otherwise be available in real-world clinical practice. In a comparison of the predictions of clinicians against common prognostication scales for 3-month functional status in real-world ICH patients, clinicians outperformed scales with regards to predictive accuracy (44).

This finding points towards the first of several limitations of prognostication scales—scales generate predictions using cohort data, yet prediction for individual patients may depend on variables not captured by scales. Furthermore, few models have been assessed for calibration (45) and robust external validation (25, 46), limiting their generalizability. Most scales were developed retrospectively, and data used to generate them include local practice patterns with regards to WLST, potentially incorporating the self-fulfilling prophesy. Finally, scales may not predict outcomes that are most important to patients and families, as the same functional outcome may lead to different perceptions of QoL for different patients. Clinicians have been shown to be poor at predicting a patient's future QoL, an inherently subjective quality, after stroke (47–49).

Despite these limitations, disclosing the results of a prognostication scale for a patient to a clinician impacts that clinician's clinical impression (50). Awareness of the limitations of scales can help ensure that the clinician utilizes these tools to complement clinical judgment rather than replace it. Recent studies suggest that making predictions based on clinical data from hospital day 5 rather than at admission may improve prognostication accuracy (51). Given the lack of objective tools for accurate prognostication and the potential for clinician bias to factor into decision-making, delaying prognostication may lead to improved prediction accuracy and clinical outcomes.

## Goals-of-Care Conversations: Determining Patients' Wishes

Besides accurate neuro-prognostication, the ideal timing of GOC discussions regarding acceptable QoL for hospitalized stroke patients requires several considerations. GOC discussions, once initiated, are often iterative (1). Prognostic information should be tailored by amount and timing to the preferences of patients and families (52).

The aim of GOC discussions should be to ascertain the patient's wishes, or best estimates thereof, in order to provide goal-concordant care. As a means to this end, ACPs and surrogate decision-makers represent two sources of information for clinicians.

### Advance Care Planning Documentation

Several types of ACPs (i.e. power of attorney, guardianship, living will, and Physician/Medical Orders for Life-Sustaining Treatment, or POLST/MOLST) exist, with variations in jurisdiction, applicability, and impact on decision-making (53). The only legally binding of these is POLST/MOLST, which serves as a standing medical order indicating a patient's wishes for treatment (54).

ACPs have variable effects on decision-making and come with several limitations. In a prospective study of hospitalized stroke patients, the presence of ACP documents and informal ACP conversations was associated with earlier transitions to CMO (55). However, other studies also specifically targeting stroke patients have suggested that the presence of ACPs does not affect clinicians' judgment for most decisions (56), implying other factors aside from ACPs play a greater role in decision-making. For instance, clinicians may endorse family members' choices for tube feeding despite contrary wishes expressed in living wills (57, 58).

Additionally, prevalence of ACPs in stroke patients is low (59); in studies of patients who died from stroke, fewer than half had completed ACPs (60, 61). Not all ACPs are readily available (61) or consistently documented (59). Though up to a quarter of strokes in the US are repeat strokes (62), ACP completion rates in stroke survivors are no different than that of the average older adult population (63). Some patients may experience financial and language barriers, as well as cultural factors, impacting ACP completion (64–66). This may also be due to the acute nature of stroke itself, making it difficult to have pre-emptive GOC conversations (67). Furthermore, ambiguous words, such as “incurable” (68) and states of “irreversible coma” are difficult to interpret in stroke (61). Most ACPs focus on specific procedures without clear-cut descriptions of scenarios pertinent to stroke (61), thus limiting their utility in determining patients' wider GOC.

### Identifying Surrogate Decision-Makers

A second source of information for clinicians to ascertain a patient's GOC is the patient's surrogate. Patients with severe stroke often do not have capacity to participate in decision-making (58, 69). Though tools such as communication boards exist to aid select intubated patients in communicating their wishes (70), the vast majority of EOL cases in the Neuro-ICU require identifying surrogates (53).

In the absence of ACPs, clinicians typically follow a prioritized list of relatives according to state law (71). When no surrogate is available, protocols may involve committees or judiciary involvement (71). Great variation exists in the use of these resources by clinicians who make decisions to limit life-sustaining treatment in the ICU, suggesting further work is needed to develop procedures in these cases (72).

## Shared Decision-Making With Surrogates: Potential Pitfalls

### Complexity of Decision-Making Factors

After surrogates are identified, several factors play a role in shared EOL care decision-making. Generally, few surrogates of critically ill patients depend solely upon clinician prognoses when estimating their loved ones' prognoses themselves (73). Many endorse being influenced by a patient's physical appearance, their faith, and their understanding of the patient's will to live in addition to, and sometimes above, the clinician's prognostication (73).

In qualitative studies, surrogates of stroke patients have endorsed reluctance in deciding to pursue CMO for loved ones (74). A recent study of nearly 800 US residents suggested that, when presented with a hypothetical scenario of a relative hospitalized with severe acute brain injury (requiring tracheostomy and PEG for survival), potential surrogates acknowledged a variety of competing concerns (75). While most surrogates prioritized respecting patients' perceived wishes and reducing suffering, surrogates may belong to different subgroups characterized by varying other top concerns: patient age, family agreement, prognostication, and cost of long-term care (75). Both non-white race and high religiosity may predict a surrogate choosing life-sustaining therapy over CMO (76). However, such a decision is still fraught with uncertainty; for example, respondents in the aforementioned study of US residents who were most concerned about cost of care were still more likely to choose tracheostomy and PEG placement over CMO compared with those less concerned (77). Clinicians must recognize that a variety of factors may influence surrogates in stroke-related GOC discussions.

### Cognitive and Emotional Biases

Potential biases exist for both clinicians and surrogates collaborating to make shared EOL decisions. Towards recommending what (if any) additional treatments to pursue in stroke patients, a clinician may be influenced, for instance, by a desire to avoid personal or legal accusations by families of patients (78) in addition to patient factors (7, 20). The clinician's prior experiences also affect these decisions; for instance, clinicians with experience in rehabilitation medicine tend to suggest continuation of life-sustaining therapy, perhaps due to a tendency to make positive prognoses (79). In these cases, clinicians may be biased towards what they would personally want in a similar situation rather than what the patient would want. In a study in which clinicians were presented vignettes of hypothetically critically-ill patients, clinician recommendations did not differ between groups who were provided the patient's values as expressed by family members vs. those who were not (80).

Several common surrogate biases warrant discussion. First, in experimental settings, surrogates' interpretations of clinician prognostications were affected by numeracy skills (81) and were often overly optimistic (82). Second, surrogates may be subject to recall bias, remembering patients as more independent than they really were prior to illness (83).

Third, surrogates may be biased by their own perceptions of acceptable QoL in contrast to patients' own wishes. In a hypothetical scenario of stroke, surrogates' ratings of a patient's QoL were not reflective of the patient's own perceptions and desire for treatment (84). Levels of patient-proxy concordance varies by decision type, with surrogates accurately predicting patient preferences for reperfusion treatment (85, 86) but not clinical trial enrollment (86). When examining withdrawal of mechanical ventilation in stroke scenarios, patient-proxy agreement varied, with lowest levels of agreement when patients wanted everything done for treatment (87). Notably, despite these discrepancies, patients continue to exhibit high levels of trust in their surrogates (87–89).

Clinicians and surrogates alike may both be subject to the “disability paradox” bias—where people with serious disabilities may report greater QoL compared to healthy individuals envisaging similar circumstances (83, 90). However, clinicians must take into consideration long-term caregiver burden and take care not to offer an overly positive prognosis that is not warranted by objective clinical data.

## Shared Decision-Making With Surrogates: Ideal Processes

Essential elements of shared-decision making models are outlined by the Agency for Healthcare Research and Quality (91, 92). A recent survey of surrogates in the Neuro-ICU showed significant room for improvement in their inclusion in decision-making and clinician communication (93). For stroke patients specifically, caregivers may not comprehend the interventions that occurred (85) and feel overwhelming uncertainty (94, 95) throughout the decisional process. Families of stroke patients tend to have relatively low satisfaction with the attention given to communication and the needs of the family despite overall high satisfaction with palliative care administration (60). Almost a third of surrogates in the Neuro-ICU experience clinically significant grief and stress reactions (96). Surrogates may feel guilty about their decisions (1, 97) and often lack time to adapt during acute stroke when rapid treatment decisions are made (74).

### Best Practices for Communication

Given these considerations, clinicians should approach the decision-making process collaboratively, negotiating the role of the clinician with surrogates (98) rather than taking a default paternalistic approach (99). Though few providers enquire about the surrogate's preferred role in decision-making (98), providers should ascertain a decision-maker's preferred level of control over EOL care decisions. Surrogates may want to make the final decision or consent to clinicians making decisions for the patient (100). Clear communication on the roles of the clinician and surrogate is key as discordancy between family members' preferred and actual decision-making roles is associated with increased depressive and post-traumatic stress disorder symptoms (101).

Our recommendations for family meetings are summarized in Table 2. Key participants to consider include interpreters (108), social workers (1, 104), spiritual care (1, 104, 109), speech therapists (110), and case managers (1, 104). Neuro-ICU nurse-led family meetings can lead to greater feelings of control by families and higher satisfaction with care (111).

**Table 2:** Recommendations for shared decision-making with surrogate decision-makers after acute stroke.

Setting the Stage for Goals-of-care	• Ensure relevant participants are involved in family meetings (i.e. patient, family, other services) (102) • Ask the surrogate decision-maker their preferences in terms of their role and that of the clinician in the shared decision-making process (98) • Utilize the ask-tell-ask approach by getting permission to present information, communicating information clearly, and checking for understanding (103)
Communicating Prognostic Uncertainty	• Acknowledge uncertainty and explain why uncertainty exists (95) • Communicate that prognosis can be altered by treatment decisions (1) • Describe possible best and worst-case scenarios of survival and future quality of life (102)
Eliciting Patient Preferences	• With open-ended questions, ask what the patient valued in life (102) (i.e. “Tell me more about what [patient] liked to do before they got sick”) (103) • Review advance care planning documents or the patient's verbally expressed wishes (1, 102)
Address Cognitive Biases	• Consider discussing common recall and/or affective forecasting biases with decision-makers (102) • Providing concrete descriptions of stroke survivors' functional outcomes after discharge may be helpful for de-biasing (83)
Ongoing Communication	• Demonstrate empathy in response to emotions (103) • Continue to assess goals-of-care over time with regular meetings (1) • Maintain consistency in communication across team members (104) and use consistent terminology to avoid confusion (105)
Consider Time-Limited Trials	• Can be used to reach consensus with families by giving patients who have a high likelihood of deteriorating a chance to respond to treatments (106) • Successful time-limited trials require defining the (1) intervention; (2) duration of intervention; (3) desired outcome; and (4) follow-up plan that may include extending the trial and pursuing or forgoing further treatment (107)

Clinicians should ensure consistent information from different providers (104, 105, 112), use an “ask-tell-ask” approach (104) and give concrete descriptions of deficits (1). Using consistent terminology avoids confusion regarding seemingly interchangeable terms such as “brain bleed”, “stroke”, and “brain hemorrhage” (105). When prognosticating, acknowledging uncertainties is important, in addition to preparing families for worst-case scenarios while using “I wish” statements to preserve hope (113). Families of stroke patients are often aware of uncertainties in prognostication but require clarification as to why such uncertainty exists (95). Given concerns of numeracy skills (81), multiple portrayals of data should be offered if quantitative estimates of prognosis are offered; risks may be perceived as higher when presented as frequencies (e.g., 1 in 10) rather than equivalent percentages (e.g., 10%) (114). Alternatively, some specialists recommend focusing on functional outcomes – with less emphasis on numerical estimates – using visual aids that illustrate the best, worst, and most likely scenarios (115). Time-limited trials can assess progress over time (106, 113) and help families come to terms with a patient's poor prognosis or manage uncertainty (Table 2).

How best to discuss prognostication and GOC after stroke remains a subject of ongoing discussion (92, 102). Decision aids, evidence-based interventions that outline the benefits/harms of decisions and their concordance with personal values (116), have been tested to assist in shared decision-making in ICUs (92, 117). A recent clinical trial using web-based decision aids for prolonged mechanical ventilation reduced surrogates' levels of decisional conflict, but did not improve prognostic concordance between clinicians and surrogates (118). Neuro-ICU-specific decision aids are currently few in number but are in development (92, 119–122). Future efforts could aim to identify different subgroups of surrogates in developing aids tailored to their priorities to facilitate shared decision-making after stroke (92).

### Expert Consultations

Traditional palliative care needs are present in over half of Neuro-ICU patients (123, 124) and consults to palliative care services are used infrequently (52, 125). Even in those who die of stroke, palliative care involvement varies greatly from 26–90% (52, 126–128). Stroke may not trigger palliative care requests from family as other diagnoses, such as cancer, might (95). Despite recognizing the importance of palliative care in stroke, clinicians may feel uncertain about when to begin addressing palliative care needs (129). As such, palliative care specialists are often only brought in during the last days of life for symptomatic management of pain, dyspnea, and mood (128, 130, 131).

It is recognized that having enough consultants to handle all palliative care needs in the Neuro-ICU may not be practical or appropriate in many situations. Palliative care consultations should not be initiated as a replacement for GOC conversations with the primary team (132). Neuro-ICU clinicians should be trained in and provide primary palliative care, including eliciting GOC and providing palliative treatments at EOL (113). However, expert palliative care consultants can help with symptom management, complicated conflict resolution, and eliciting further patient values/needs (133). Current palliative Neuro-ICU screening tools (123) and new models of palliative care delivery (102, 134–137) are being explored to assist clinicians with thresholds for consulting expert palliative care.

Conflicts may occur surrounding decisions of artificial nutrition/hydration (60, 74, 112, 138), resuscitation (112), and care transitions (112), particularly when impressions of prognosis are different between surrogates and clinicians despite multiple attempts at family conferences (52, 138, 139). Though protocols differ, ethics consultations can help resolve conflicts between decision-makers and providers (140). Should providers believe that inappropriate treatment has been requested, a series of steps are recommended for conflict resolution by the American Thoracic Society (141, 142).

## Conclusion

In this brief review, we discussed factors to consider when engaging in EOL decision-making, including prognostication, determining patient wishes, and interacting with surrogates with the goal of shared decision-making. It is important to note that even after decisions to WLST, families require ongoing support (95). Expectations must be discussed after WLST, which does not always mean imminent death (1, 143), as families often expect death early on and are distressed by prolonged dying processes (138).

## Author Contributions

LG, CZ, and DH all discussed the content of this manuscript in its planning stages. LG and CZ drafted this article, with subsequent revisions from DH. All authors contributed to the article and approved the submitted version.

## Conflict of Interest

DH has received philanthropic funds from the Apple Pickers Foundation (Westerly, RI) for pilot testing of decision aids for families of patients with severe acute brain injury. The remaining authors declare that the research was conducted in the absence of any commercial or financial relationships that could be construed as a potential conflict of interest.

## Publisher's Note

All claims expressed in this article are solely those of the authors and do not necessarily represent those of their affiliated organizations, or those of the publisher, the editors and the reviewers. Any product that may be evaluated in this article, or claim that may be made by its manufacturer, is not guaranteed or endorsed by the publisher.
